# Covid-19 and Priorities for Research in Aging

**DOI:** 10.1017/S0714980820000331

**Published:** 2020-09-02

**Authors:** R. J. Rylett, Flamine Alary, Joanne Goldberg, Susan Rogers, Patricia Versteegh

**Affiliations:** 1 Robarts Research Institute, University of Western Ontario, London, Ontario; 2 Centre de recherche de l’Institut universitaire de gériatrie de Montréal, Université de Montréal, Quebec

**Keywords:** vieillissement, Instituts de recherche en santé du Canada, Institut du vieillissement, priorisation de la recherche, adultes plus âgés, Covid-19, aging, Canadian Institutes of Health Research, Institute of Aging, research prioritization, older adults, Covid-19

## Abstract

This article describes priority areas for research on the impact of the Covid-19 pandemic on older adults that have been identified by the CIHR Institute of Aging (CIHR-IA). The process used by CIHR-IA consists of several iterative phases and thus far has resulted in identification of three key areas for Covid-19 research needs and four cross-cutting thematic areas. The key research priority areas are as follows: response of older adults to disease, vaccination, and therapeutics; mental health and isolation; and supportive care environments. The four cross-cutting themes are equity, diversity, and inclusion (EDI); ethical/moral considerations; evidence-informed practices; and digital health technologies. The priorities outlined in this article will inform CIHR-IA’s responses to Covid-19 research needs.

## RÉSUMÉ

Cet article présente les domaines prioritaires de recherche sur les impacts de la pandémie de COVID-19 chez les personnes âgées telles qu’ils ont été identifiés par l’Institut du vieillissement des IRSC (IV-IRSC). Le processus utilisé par l’IV-IRSC a comporté plusieurs phases itératives qui ont permis d’identifier trois secteurs prioritaires parmi les besoins de la recherche relative à la COVID-19, et quatre axes thématiques transversaux. Les secteurs de recherche prioritaires sont (1) la réponse des personnes âgées à la maladie, à la vaccination et aux traitements, (2) la santé mentale et l’isolement, et (3) les milieux de soins soutenants. Les quatre thèmes transversaux sont (a) l’Équité, la diversité et l’inclusion (EDI), (b) les considérations éthiques et morales et (c) les pratiques fondées sur les données probantes, et (d) les technologies numériques de la santé. Les priorités décrites dans cet article guideront les réponses de l’IV-IRSC aux besoins de la recherche sur la COVID-19.

## Background

On March 11, 2020, the World Health Organization announced that Covid-19 had reached pandemic status.^1^ Soon after, research activities across Canada changed dramatically, with laboratories and research facilities closing and most researchers and trainees adapting their investigations to work from home where possible. It became apparent that, coincident with these changes, older adults were disproportionately adversely affected by Covid-19.

The mission of the Canadian Institutes of Health Research Institute of Aging (CIHR-IA) is to support research, to promote healthy aging, and to address the causes, prevention, screening, diagnosis, treatment, support systems, and palliation for the complex health challenges that can be present in older individuals.^2^ CIHR-IA promotes research programs and other research enabling activities to identify and address the knowledge gaps and opportunities related to promoting the health and wellness of Canada’s aging population.

CIHR-IA focuses on the entire trajectory of life while specifically addressing the health challenges of older individuals and is a national leader in addressing health research priorities related to aging. Initiatives led by CIHR-IA not only link and support researchers across the country, but also bring together a broad group of stakeholders, including different levels of government, practitioners, health charities, voluntary health organizations, and older adults themselves. At the start of the Covid-19 crisis, CIHR-IA mobilized quickly to identify research priority areas related to this pandemic and its dramatic effects on older adults. The goal of this article is to describe the approach implemented by CIHR-IA to rapidly understand Covid-19 research needs for older adults in Canada. These priorities will be used to shape future actions of CIHR-IA within the context of Covid-19.

## Actions and Consultations

**Figure 1:**
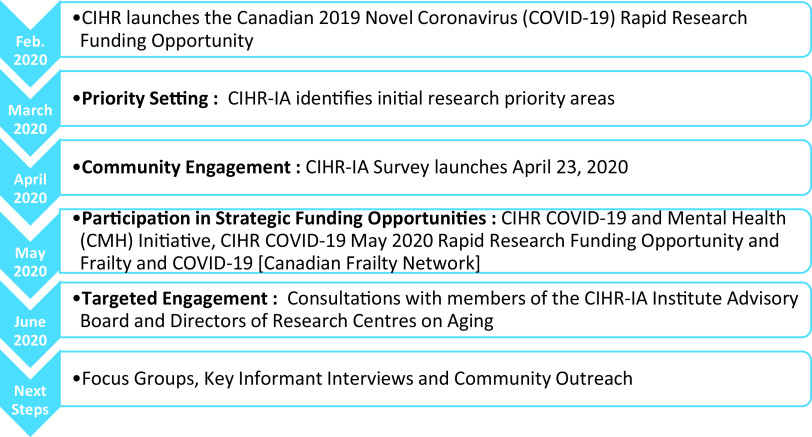
Timeline for the identification of research priority areas on the impact of the Covid-19 pandemic on older adults

The process initiated by CIHR-IA consists of a number of actions and consultations as illustrated in Figure 1 and described below.

In early February 2020, CIHR launched the Canadian 2019 Novel Coronavirus (Covid-19) Rapid Research Funding Opportunity. A total of 100 grants were funded in this competition for a Government of Canada investment of $55.3 million, with the primary focus on medical countermeasures research and social and policy countermeasures research. At this early stage in the identification and investigation of the health impacts of Covid-19, older adults had not yet been recognized as an especially vulnerable population. Thus, no funded projects focused on Covid-19 consequences on the health, wellness, and welfare of older adults. Given this outcome and the rapidly evolving situation related to Covid-19 and older adults, CIHR-IA mobilized to identify research priorities to respond to the needs of the aging population in preparation for the Institute’s participation in funding opportunities coming forward from CIHR and partners.

With review of the available published literature and emerging data, CIHR-IA identified initial areas of research need related to Covid-19’s impact on older adults. This was followed by the initiation of a fast-track process to engage stakeholders and gain insight into the impact and implications of Covid-19 on research in aging in Canada. In April 2020, CIHR-IA sent a survey to the more than 4,000 mailing list subscribers; over 600 responses were received from researchers, trainees, decision-makers, health care administrators, public servants, health care providers, older adults, and caregivers. The survey comprised eight questions designed to obtain both quantitative and qualitative responses. Qualitative responses were analysed using a thematic approach and classified by emerging themes. In early June, CIHR-IA engaged in targeted consultations with key stakeholders, including the Institute of Aging Advisory Board members and the directors of the 35 Canadian Research Centres on Aging. Information from these consultations was collated with data obtained from the survey, resulting in the identification of several key research priorities.

## Key Research Priorities

**Figure 2:**
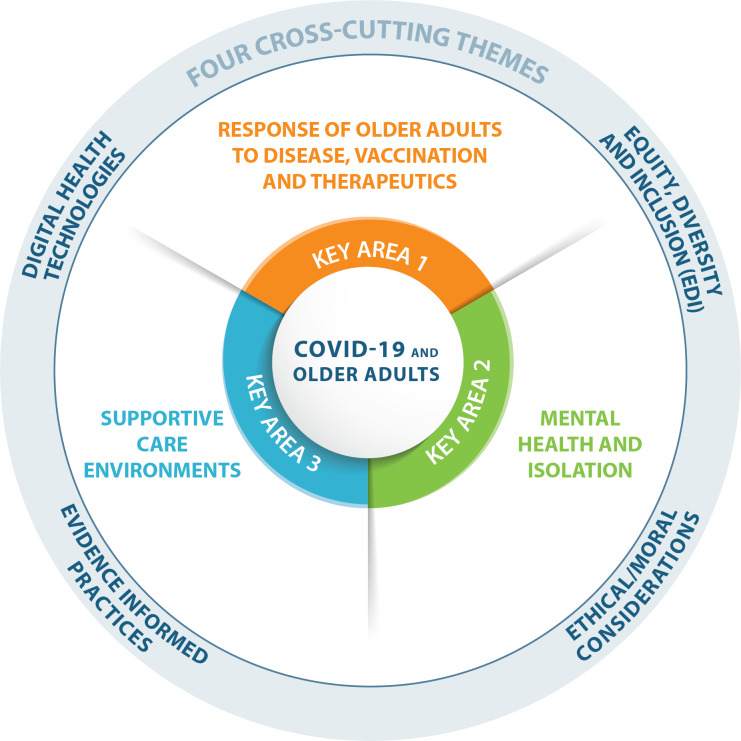
Infographic depicting key areas of research priority and cross-cutting themes

The CIHR-IA priority identification process resulted in three key areas and four cross-cutting themes for research on the impact of Covid-19 on older adults and aging, as illustrated in Figure 2.

### Key Area 1: Response of Older Adults to Disease, Vaccination, and Therapeutics

Aging is a multifaceted process involving numerous cellular and molecular mechanisms in different organ systems. This can include functional and structural alterations in the immune system that can manifest as a decreased ability to fight infection and constitutive low-grade inflammation (inflammaging) (Franceschi & Campisi, 2014). Immunosenescence and inflammaging factors are involved in a range of age-related diseases and correlates with poor response to vaccination (Keenan & Allan, 2018), with this being an important consideration in the development of effective vaccination strategies for older adults. There is also no information about the impact of coronavirus on body systems in older adults and their increased susceptibility to Covid-19–related sequelae. A geroscience approach (Kennedy et al., 2014) could provide much-needed information on the interaction involving the aging process, the development of chronic diseases, and the impact of the coronavirus on physiological systems.

The current development of therapeutics for Covid-19 and clinical trials may not adequately consider the potential impact of aging. Older adults can have altered metabolism and disposition of drugs, as well as enhanced drug sensitivities and interactions (Mallet, Spinewine, & Huang, 2007). These adults may also be taking multiple medications related to chronic health conditions, presenting polypharmacy challenges upon the administration of Covid-19 therapeutics. Thus, older adults constitute a special-needs population for treatment and should be adequately represented in clinical trials for drugs/treatments and vaccines.

Examples of research needs in Key Area 1:

- Investigations to address the primary and secondary impact of coronavirus on body systems and co-morbidities.
- Research to understand the effect of the coronavirus on the aging immune system to inform the development of vaccination strategies. Vaccine design and vaccination approaches need to be tailored for older adults to ensure efficacy, safety, protection, and healthy aging.
- Therapeutic trials that ensure that older adults of both sexes are included since men and women age differently and do not always respond similarly to treatments.
- Initiatives for equitable access and appropriate dosing of therapeutics and vaccines to protect at-risk populations that specifically include consideration of the needs of older adults.

### Key Area 2: Mental Health and Isolation

Older adults are experiencing increased stress and mental health challenges resulting from their increased vulnerability to the virus and the strict isolation requirements that have been imposed during the coronavirus crisis (Chu, Donato-Woodger, & Dainton, 2020; Steinman, Perry, & Perissinotto, 2020). Recreation and stimulation programs in residential living and long-term care homes have also been temporarily discontinued further increasing isolation and loneliness. Indeed, the isolation may be worse for older individuals living alone because caregivers cannot visit, and many home care services and community programs for older adults and their caregivers, such as adult day programs, have been suspended leading to loss of support (Flint, Bingham, & Iaboni, 2020). Many older adults have been placed in positions where they have less autonomy in decision-making regarding their own care and independence. This has led to a sense that they have lost the ability for self-determination and “mattering” (Flett & Heisel, 2020); that is, they have lost the sense of being valued or of having a voice. Being able to make or participate in making decisions about their own well-being is predictive for protecting both mental and physical health of older adults.^3^ This is crucial for decisions about the care and provision of medical resources to the frail elderly who have contracted Covid-19 (Hubbard et al., 2020). Ageism – a discriminatory attitude toward people of advanced age – may have contributed to detrimental effects on the health and longevity of older adults with Covid-19 (Fraser et al., 2020).

Examples of research needs in Key Area 2:

- Development and assessment of tools to reduce anxiety and mitigate social isolation with intervention programs. These could include, among others, the development of readily accessible and user-friendly digital health care solutions to keep isolated older adults socially connected. Educational virtual programs or platforms could be designed for health professionals and workers as learning tools on how to cope with stress and to protect their own health and the health of those for whom they care.
- Research to assess the impact of isolation on cognitive function and the decline in dementia, coupled with the development and delivery of interventions. This can include the development of tools for remote monitoring of health and cognitive function.
- Evidence-informed collaboration with health care policy-makers to develop clinical guidelines for the provision of care to older adults with Covid-19 when health care services and resources limited. In particular, the potential rationing of health care services with older adults given lower priority can negatively impact their mental health and that of their family and caregivers.

### Key Area 3: Supportive Care Environments

Older adults live in many residential settings with varying levels of support. The health and social crises resulting from Covid-19 have placed a spotlight on issues that have existed for years in the care delivery system for older adults. Although the pandemic has affected older adults across Canada regardless of their living arrangements, no part of this system has been impacted more profoundly than have long-term care (LTC) homes.^4^ In Canada, 80 per cent of the deaths due to Covid-19 occurred in LTC homes, with this being the highest percentage of any reporting country worldwide^5^ (Estabrooks, Flood, & Straus, 2020; Estabrooks, Straus, et al., 2020). However, only about 7 per cent of older adults in Canada reside in LTC homes,^6^ with most of the older adults living in the community and requiring support from home and community care services or family members to allow them to function relatively independently.^7^ Quarantine or social distancing have seriously impacted the ability of these support systems to provide essential care and resources to older adults living independently, leaving them alone, increasingly vulnerable and unable to obtain the basic needs for survival. Moreover, many older adults have one or more medical conditions requiring access to health care services and treatments that may be interrupted or inaccessible during the pandemic. This could potentially be exacerbated by inequities in the medical system leading to loss of critical care for older adults.

Examples of research needs in Key Area 3:

- Research that informs best practices for care in residential settings for older adults. This includes developing and implementing evidence-based guidelines for infection control, workforce and staffing, and care of vulnerable populations.
- Assessment of the needs and evaluation of implementation plans for providing community-based care and services to older adults living independently or in residential settings in the post-pandemic recovery period.
- Development of programs and policy for delivery of health care to older adults in a manner that is sensitive to their special needs, ensuring that they are included in the decision-making process. This includes the provision of and access to remote care to address physical and mental health needs.

## Cross-cutting Themes and Key Drivers in the Areas of Ethics, Health Services, Public Policy, and Population Health

Four important cross-cutting themes are also identified that intersect with the aforementioned three key research areas.

- **Equity, diversity, and inclusion (EDI)**
  CIHR has implemented a range of strategies and solutions to foster equity, diversity, and inclusion in the research ecosystem.^8^ It is crucial during this Covid-19 crisis for investigators carrying out research on aging to consider Indigenous and other under-represented populations, socio-economic and cultural factors, equitable access to health system resources, and patient engagement, particularly as these factors relate to older adults.
- **Ethical/moral dilemmas around end-of-life care in pandemic situations or emergencies**
  Ethical and moral considerations in research on older adults are critical within the Covid-19 crisis. Research related to illness, resource rationing, palliative care, and isolation are of critical importance.
- **Trials informing best practices for care of older adults with covid-19 and translation and implementation of knowledge for best practices in emergency preparedness**
  CIHR-IA supports the inclusion of older adults in trials that inform best practices that involve Covid-19 care. Historically, older adults have been omitted from trials that will impact their own care.
- **Digital health technologies supporting health services and care delivery, including drug discovery and vaccine production, and monitoring and social interventions**
  CIHR-IA is a co-lead of the CIHR eHealth Innovations initiative. This initiative supports exceptional national and international digital health research to develop solutions to maintain and improve the lives of older adults. Research that brings together a diversity of stakeholders will enable enhanced collaboration through the entire health research continuum from basic research to public health.

## Summary and Conclusions

Soon after the start of the Covid-19 pandemic, it became apparent that older adults were the most at-risk group to develop complex symptoms and had the greatest risk of mortality. To advance our mandate to improve the health and lives of older adults, CIHR-IA reacted rapidly, including by consulting stakeholders and experts, to identify priority research needs related to older adults and Covid-19. The key research areas laid out in this article, ranging from the response of older adults to disease, vaccination, therapeutics, and clinical trials, to mental health and supportive care environments, will continue to inform the responses made by CIHR-IA over the longer term.

In parallel with the consultation process and identification of priority research needs, CIHR-IA rapidly mobilized to partner on two important CIHR funding opportunities; Covid-19 May 2020 Rapid Research Funding Opportunity and the Covid-19 and Mental Health (CMH) Initiative, as well as a funding opportunity initiated by the Canadian Frailty Network (CFN) to fund research projects focused on the interaction between frailty and Covid-19 in older adults. Research carried out in these three programs has the potential to begin to address the priority needs in each of the key areas identified. CIHR-IA will continue to seek opportunities to develop and partner on research programs that will address the needs of older adults in this area.

The four cross-cutting themes identified through the consultation process pinpoint crucial elements to be included in research on the impact of Covid-19 on older adults. As with all health research, considerations related to equity, diversity, and inclusion of under-represented populations must be integrated into the research plan and, in particular, older adults must be engaged meaningfully in all aspects of the research. With regard to the specific situation of older adults during the Covid-19 pandemic, considerations related to mental health issues and ethical/moral dilemmas around end-of-life care in a pandemic or emergency situation should be carefully integrated into the research. This is an opportunity to rethink the status and role of older adults in our society and to specifically address the impact and influence of ageism in decision-making and the delivery of care. Research leading to public policies that reduce social exclusion of seniors and fight ageism and stigma associated with end of life, frailty, and vulnerability, is needed now more than ever. The inclusion of older adults in trials to inform best practices for Covid-19 care is of the utmost importance. At this time of evolving tools and techniques, digital health research should be harnessed to bring solutions to maintain and improve the lives of older adults.

CIHR-IA seeks to ensure that research needs on aging in Canada are met and so is committed to fostering research programs to find approaches to support the health and wellness of older adults during the Covid-19 pandemic and in the post-pandemic recovery period.

## Notes

1
https://www.who.int/dg/speeches/detail/who-director-general-s-opening-remarks-at-the-media-briefing-on-covid-19---11-march-2020

2 CIHR Institute of Aging Strategic Plan 2019-2021. Living Longer, Living Better. https://cihr-irsc.gc.ca/e/46837.html

3
https://news.yorku.ca/2020/06/16/feeling-expendable-during-the-pandemic-why-older-adults-need-to-feel-like-they-matter/

4
https://www.canada.ca/en/health-canada/services/home-continuing-care/long-term-facilities-based-care.html

5
https://www.cihi.ca/en/long-term-care-and-covid-19-international-comparisons

6
https://www.statcan.gc.ca/eng/subjects-start/seniors_and_aging

7
https://www.canada.ca/en/health-canada/services/home-continuing-care/home-community-care.html

8
https://cihr-irsc.gc.ca/e/50068.html
